# Working Toward Integrative Health Equity: Reflections from Acupuncture Implementation Within a Pediatric Primary Care Safety-Net Clinic

**DOI:** 10.1089/imr.2021.0016

**Published:** 2022-05-04

**Authors:** Jenifer Matthews, Karen Villanueva, Susan Yee, Ariana Thompson-Lastad

**Affiliations:** ^1^ UCSF Benioff Children's Hospital Oakland, Oakland, CA, USA.; ^2^ UCSF Osher Center for Integrative Medicine, San Francisco, CA, USA.; ^3^ UCSF Department of Family and Community Medicine, San Francisco, CA, USA.

**Keywords:** pediatrics, acupuncture, health equity

Complementary and integrative health (CIH) care is well positioned to provide culturally affirming, patient-centered, and evidence-informed care for vulnerable children and families. Our pediatric primary care clinic has responded to the growing evidence base for CIH in pediatrics and the multiple national calls to action to include nonpharmacologic approaches in the treatment of pain by implementing acupuncture.^1,2^ Our clinic, a Federally Qualified Health Center (FQHC) in Oakland, CA, USA, serves a multiracial low-income pediatric patient population with 91% of the patients insured by Medicaid.

Families served by this clinic face high levels of community trauma and structural violence, contributing to high prevalence of anxiety, depression, post-traumatic stress disorder, and chronic and acute pain conditions.^3,4^ Within the current biomedical paradigm, treatment options often include pharmaceuticals, lifestyle recommendations, and mental health referrals, but access to these can be fragmented and frustrating for both patients and providers alike. In their call to action, “Integrative Medicine and the Imperative for Health Justice,” authors Chao and Adler urge us to “leverage integrative medicine to advance health equity, that is, the attainment of the highest level of health for all people.”^5^

Acupuncture is one form of CIH that has a growing evidence base for use among children. There is strong interest in the use of pediatric acupuncture in the United States, with evidence for the safety and feasibility of pediatric and adolescent acupuncture as well as acceptability of acupuncture by children.^6,7^ The benefits of acupuncture for children have been documented in children experiencing chronic pain chemotherapy-associated symptoms, and anxiety, among other conditions.^8–10^ Widespread implementation of acupuncture has been limited for multiple reasons, including limited or nonexistent insurance reimbursement as well as assumptions that children will be fearful of needles.

The term “acutherapy” was introduced in the clinic to encompass acupuncture, acupressure, and Gua Sha. This term was used due to concern about patients’ reactions and negative conditioning to the term “puncture” and the potential association of an acupuncture needle and treatment being similar to puncture with a hypodermic needle for venipuncture or vaccine administration. Pediatric patients and their families, particularly those who are unfamiliar with this form of care, can be wary when they hear the words, “puncture” or “needles,” and language matters with all patients, especially those who have a history of chronic illness or medical trauma.

As part of a larger hospital-based interdisciplinary Integrative Medicine Medical Staff Committee, the group—including a physician, licensed acupuncturist/nurse, social worker, and sociologist—collected preliminary data to better understand staff and family priorities, as well as potential barriers to care as acutherapy was implemented in this clinic. Although there are exemplary models of pediatric integrative medicine implementation, these programs are often in academic hospital-based inpatient settings and it can be difficult to translate these experiences to a pediatric primary care safety-net setting.^11–13^ However, Silver Highfield *et al.* found that by reducing barriers to access, both pediatric and adult patients in a similar safety-net urban setting utilized acupuncture as part of ongoing primary care.^14^

To reduce barriers and center family voices in the planning stage of this project, caregivers were surveyed about their current CIH usage and interest, and offered caregiver engagement sessions in multiple languages in the clinic. Over a 3-month period in 2019–2020, families were surveyed in three languages—English, Spanish, and Arabic—about current CIH usage and potential interest in CIH modalities if offered in the clinic, as well as ways to make CIH offerings convenient and accessible. Fifty-nine caregivers completed the survey, and included families broadly reflective of the patients served in clinic* (Table 1).

**Table 1.** Caregiver Demographics

Primary language^a^, % (*n*)	
English	76 (45)
Spanish	24 (14)
Arabic	5 (3)
Tigrinya	2 (1)
Other	5 (3)
Insurance^b^, % (*n*)	
Medicaid	88 (52)
Private insurance	12 (7)
No insurance	0 (0)
Ethnicities^c^, % (*n*)	
African American or Black	39 (23)
Latino/Hispanic	32 (19)
White/Caucasian	17 (10)
Asian	12 (7)
Other	12 (7)
Native American/	3 (2)
American Indian	
Pacific Islander/Samoan/Native Hawaiian	2 (1)
Children's ages (years)^d^, % (*n*)	
0–2	29 (17)
2–5	41 (24)
6–11	46 (27)
12–18	30 (18)
>18	5 (3)

Survey participants could select multiple response options as applicable to their identities and situations

^a^ Survey question: What are the primary languages you speak at home?

^b^ Survey question: What kind of insurance does your child(ren) have?

^c^ Survey question: What is your race or ethnicity? (Check all that apply.)

^d^ Survey question: How old are your children? (Check all that apply.)

English (76%), Spanish (24%), and Arabic (5%) were the primary languages spoken at home. Most caregivers identified Medicaid (88%) as their child's insurance and self-identified as African American or Black (39%), Latino/Hispanic (32%), or White/Caucasian (17%). Almost one-fourth of children were identified by their caregivers to have a chronic illness.

Of the 59 caregivers surveyed, over half (53%) had used CIH approaches for their children for a wide range of health conditions, most commonly anxiety/stress (29%), pain (28%), and asthma or allergies (26%). The most common forms of CIH used by families were herbal medicine/supplements (29%), mindfulness/meditation/relaxation or breathing practices (27%), yoga/Tai Chi/*qigong* (15%), and exercise and nutrition coaching (15%).

Over half of families were interested in CIH being offered in the primary care clinic with exercise and nutrition coaching (58%), mindfulness/meditation, relaxation and breathing training (51%), and acutherapy (47%) as the top three priorities. When asked specifically about making acutherapy accessible for their children, most caregivers indicated that this clinic should work to make the service free or covered by insurance (82%) as well as making sure clinic staff were informed and knowledgeable about CIH (52%).

In addition, informal engagement sessions were offered for caregivers who completed the survey to learn more about integrative health modalities and to share what resources and practices they use. These sessions were hosted to create a space to affirm families’ healing practices and gather input into clinic design and offerings. Notes on caregivers’ comments were taken in each session. Caregivers’ perspectives on CIH focused on four areas:

1. Interest in increasing personal understanding of CIH, specifically access to research findings about CIH modalities and how they work with conventional medicine.
2. Desire for CIH implementation and trainings in the clinic, including approaches that would affirm and include families’ existing cultural and spiritual healing practices. One African American participant initially stated, “I don't have any cultural practices. Those were lost,” then realized after looking at other parents’ practices, that she, too, had healing practices that had been shared with her by her grandmother and mother.
3. Emphasis on the need for CIH to be accessible to all patients, by ensuring well-trained staff, coordinated care, low-cost or no-cost services, and expanded hours.
4. Shared perspective on CIH as an opportunity to create a more collaborative and less hierarchical approach to primary care. Caregivers suggested that CIH implementation might make care more collaborative and that this could promote healing and understanding between families and staff.

Building on this information, acutherapy began being offered at no cost to clinic patients in January 2020 through a shared-visit model, where patients were seen by both a physician and a licensed acupuncturist with the physician billing for the visit. The licensed acupuncturist's time was paid for through a hospital-based innovation grant. The COVID-19 pandemic required this clinic to discontinue all nonurgent in-person visits after seven acupuncture clinic sessions. In-person acutherapy visits resumed in Fall 2020. During a 6-month period (September 2020 through February 2021), the licensed acupuncturist completed 102 patient visits with patient demographics reflective of this clinic.

The most common diagnoses were chronic pain related (including primary pain syndromes, pain secondary to traumatic injury, and congenital concerns with pain-like osteogenesis imperfecta), as well as anxiety/depression, and orthopedic-related concerns (temporomandibular joint dysfunction, spasticity related to cerebral palsy, and arthrogryposis). The age range of patients was 12 months to 22 years, with 75% of patients being 12 years or older. Teen patients were especially enthusiastic and open to trying acupuncture for the first time.

For younger patients, “acutherapy” was often introduced in the form of acupressure and caregivers were taught how to give acupressure at home. The licensed acupuncturist's time continues to be supported through philanthropic support and the physician has also been trained in medical acupuncture and has begun offering services as well, expanding access for families.

This pilot study reinforced the need to address the barriers many families face to access CIH, including acupuncture. Caregivers in this clinic indicated that the most important barrier to acupuncture access is cost/insurance coverage, and similar concerns exist for other integrative modalities. Challenges with implementation and sustainability of acupuncture and other integrative modalities in a safety-net clinical setting similar to the authors include lack of insurance reimbursement. There are policy changes that indicate progress, such as Medicare for the first time reimbursing for acupuncture for low back pain. This clinic offers acutherapy through a shared-visit model for patients, therefore, requiring no out-of-pocket cost, and found a positive response from families with a growing demand for services.

Acupuncture can be a powerful and evidence-based healing modality for pediatric populations in safety-net settings. In efforts to bring acutherapy and CIH to patients, one must be careful not to make assumptions about the patient and family interests, skills, and knowledge base. By forming equitable partnerships with integrative, traditional, and indigenous practitioner colleagues and patients, one can build a foundation in which to understand the needs, priorities, and desires of each stakeholder.

By working together with respectful collaboration and humility, the team found that it was able to provide a unique healing experience for patients and families as well as create a nourishing clinical environment for ourselves. Implementation of whole medical systems like Traditional Chinese Medicine into a safety-net clinic model can be successful through respectful engagement with patients and colleagues as partners in the planning, implementation, and evaluation of new models of care.

## Abbreviation Used

CIH: complementary and integrative health

## Notes

^*^
Study was approved by UCSF Benioff Children's Hospital Oakland Institutional Review Board.
